# The protective effects of cognitive empathy and emotional empathy on gambling disorder are mediated by risk aversion and responsible gambling attitude

**DOI:** 10.1186/s12888-024-05509-5

**Published:** 2024-01-23

**Authors:** Hui Zhou, Anise M. S. Wu

**Affiliations:** 1 Department of Psychology, Faculty of Social Sciences, University of Macau, Macao, China; 2 Centre for Cognitive and Brain Sciences, Institute of Collaborative Innovation, University of Macau, Macao, China

**Keywords:** Empathy, Gambling disorder, Attitude, Risk aversion, Responsible gambling

## Abstract

**Background:**

Based on social cognitive theory, this study aimed to examine whether and how social abilities (i.e., cognitive empathy and emotional empathy) are associated with gambling disorder (GD) by incorporating attitudes toward general risk (i.e., risk aversion) and responsible gambling as potential mediators of this link.

**Methods:**

A convenience sample of 580 past-year lottery gamblers (*M*_*ag*e_ = 34.07, *SD* = 13.36; 50.4% female), recruited near lottery sales shops, completed an anonymous paper-version questionnaire on site. Data were collected using the DSM-5 diagnostic criteria for GD, Interpersonal Reactivity Index, Risk Aversion Scale, Positive Play Scale, and demographic items. Path analysis and mediation analysis were applied to examine the effects of cognitive empathy and emotional empathy on GD and the mediating roles of risk aversion and responsible gambling attitude.

**Results:**

Our results showed that cognitive empathy, but not emotional empathy, was significantly and negatively correlated with GD. Also, the effect of cognitive empathy on GD was fully mediated by risk aversion and responsible gambling attitude, whilst the total indirect effect of emotional empathy on GD was nonsignificant. As hypothesized, the indirect paths from both types of empathy to GD were significantly and serially mediated by risk aversion and responsible gambling attitude.

**Conclusion:**

Cognitive empathy, distinct from emotional empathy, was a statistically significant correlate of GD. Moreover, the path model results also suggest that responsible gambling attitude was a salient protective factors against GD. Future GD prevention efforts may benefit from paying more attention to the role of responsible gambling attitude.

## Introduction

Gambling disorder (GD), as the only behavioral addiction listed officially as a mental disorder in the fifth edition of the Diagnostic and Statistical Manual (DSM-5) [1], is marked by an excessive and maladaptive gambling pattern with addictive symptoms, such as preoccupation and withdrawal. Individuals with GD commonly report functional impairments in not only intrapersonal but also interpersonal domains (e.g., interpersonal conflicts and poor social relationships) [1, 2], and their parents, spouse, other family members and friends, as well as communities are adversely affected [3, 4]. This study, based on social cognitive theory [5], aimed to examine whether and how individuals with two types of empathic ability (i.e., emotional empathy and cognitive empathy) are at reduced risk of developing GD because of their negative attitudes toward general risk (i.e., risk aversion) and responsible gambling attitude.

### Empathy and GD

Empathy, the ability to experience and understand what others feel [6], consists of two components (i.e., emotional empathy and cognitive empathy) [6, 7] and contributes to adaptation in one’s environment [8, 9]. Individuals with emotional empathy are able to detect the emotional cues of others, which allows them to automatically sense or tune into what others are feeling, whereas cognitive empathy refers to the ability to understand others’ thoughts and emotions by adopting their perspectives [6, 10, 11]. Thus, individuals lacking empathic ability tend to have difficulty feeling others’ personal emotions and understanding others’ thoughts. As an ability that plays an integral role in interpersonal interactions and social functioning [12], empathy is generally associated with not only better mental and social wellbeing (e.g., personal accomplishment and life/relationship satisfaction) [13–15] but also fewer mental problems or disorders (e.g., burnout, autism, and antisocial personality disorder) [15–17].

Concurrently, there is good evidence for the association between empathy and addiction. For example, lower levels of both emotional empathy and cognitive empathy have been consistently reported in individuals with substance-related addiction (e.g., alcohol/drug use disorder) [18–21]. Compared with substance-related addiction, empathy and GD have been less studied [22], and only two empirical investigations have explored their association. According to the findings of one study, disordered gamblers showed abnormal levels of both cognitive empathy and emotional empathy via self-report measure and worse performance in a perspective-taking task when compared with their healthy counterparts [23]. In another recent fMRI study, disordered gamblers show altered effective connectivity between brain networks of empathy and gambling compared to healthy controls [24]. Consistent with the findings of previous studies, we hypothesized negative correlations between these two types of empathy and GD (Hypothesis 1).

### The roles of risk aversion and responsible gambling attitude

Whereas empathy and GD have been shown to be related in two studies, the psychological mechanisms underlying this association have not been empirically investigated. Bandura [25, 26] has proposed that individuals’ behaviors are largely associated with their cognitive processing and conscious reasoning. Indeed, his social cognitive theory [5] further clarify the important role of cognitive factors such as attitudes in determining a behavior. For example, individuals’ expectation and evaluation of the possible behavioral outcomes (i.e., attitude) affect their behaviors. Social cognitive theory has been useful in understanding substance-related addition (e.g., alcohol use disorder) [27] and behavioral addiction (e.g., GD and networking addiction) [28–30]. It was also used as a guide in the present study to better understand the potential link between both types of empathy (i.e., cognitive and emotional) and GD via attitudes toward risk and/or responsible gambling.

Risk aversion has been defined as a generalized pattern of negative attitudes toward potential risk from various outcomes in life [31]. Individuals with high levels of cognitive empathy and emotional empathy may tend to avert risk, probably because they may be more sensitive to others’ negative emotions [32, 33] in response to their failures than any positive emotions that might be associated with their wins. Aversive affective experiences, would, consequently, facilitate the development of negative attitudes toward risk and risk-associated objects and events. The findings of Santesso and Segalowitz [34] supported this premise by showing that self-reported empathy was significantly and positively correlated with the amplitude of error-related negativity, which indicated ones’ sensitivity to the negative consequences of decisions [35]. Therefore, we hypothesized that both cognitive and emotional empathy would be positively correlated with risk aversion (Hypothesis 2).

Despite the scarcity of research, risk aversion is a potential protective factor against GD. Lower sensitivity to risk and preferred risky choices in the Iowa Gambling Task among disordered gamblers have been consistently reported in previous studies [36]. Moreover, neuroimaging studies have shown that individuals with GD had an abnormal preference for risk, with altered activities in reward systems in money-related decision-making tasks [37–40]. Whereas all these previous studies investigated the link between ones’ sensitivity to money-related risk with GD during decision-making tasks, this study aimed to examine the association between negative attitudes to general risk and GD. We hypothesized a negative correlation of risk aversion with GD (Hypothesis 3). Considering its hypothesized correlation with empathy, we also hypothesized the mediating effect of risk aversion in the associations between two types of empathy and GD (Hypothesis 4).

In addition to attitudes toward general risk, this study also considered responsible gambling attitude, which is a specific type of attitudes toward gambling, referring to how one evaluates a controlled and responsible pattern of gambling behavior (e.g., thinking that one should be aware of his/her gambling amount and gambling is not a good way to make money) [41]. Consistent with social cognitive theory, previous studies showed that people with responsible gambling attitude were more likely to report more regulatory behaviors over their gambling and were less likely to experience GD symptoms [41, 42]. A negative correlation between responsible gambling attitudes and GD was also hypothesized in this study (Hypothesis 5).

Cognitive empathy and emotional empathy may also facilitate an individual’s responsible gambling attitude. For examples, a gambler may develop more favorable attitudes toward responsible gambling if he/she detects and feels the negative emotional responses of family members’ toward uncontrolled gambling via emotional empathy, as well as adopts their perspectives on gambling (e.g., disapproval of large and frequent betting) via cognitive empathy. Indeed, gamblers with high levels of empathy have a better sense of the socially accepted attitude toward gambling (e.g., gambling is for entertainment but not money-earning) [43]. Cognitive empathy has also been shown to be involved in assessing others’ intentions and beliefs/attitudes [44–46] before the internalization of such beliefs/attitudes can happen [47]. Thus, the study hypothesized positive correlations between both emotional and cognitive empathy and responsible gambling attitude (Hypothesis 6). Considering its hypothesized link with GD, this study also hypothesized that responsible gambling attitude mediated the associations between the two types of empathy and GD (Hypothesis 7).

**Fig. 1:**
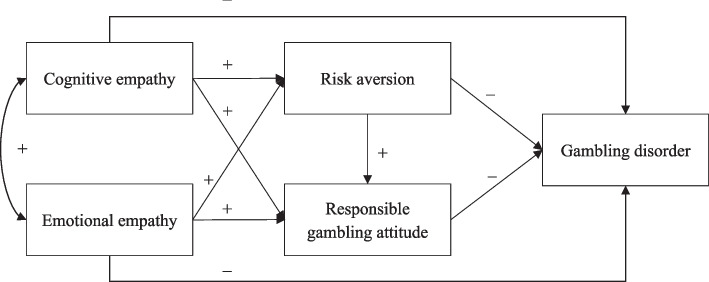
The hypothesized path model

More general cognitions are commonly linked with individuals’ appraisal of a related event, taking gambling for example [48], and a previous study has found a positive correlation between risk propensity (i.e., a personal attribute that inclines one to take risk) and irrational gambling cognition (e.g., having more gambling-related knowledge and skills than others) [49]. Gamblers who are risk averse may be disposed to consider gambling as an activity characterized by “chance” and “risk” and adopt responsible gambling attitude. We, therefore, hypothesized a positive correlation between risk aversion and responsible gambling attitude (Hypothesis 8). Considering the empathy-attitude-GD link aforementioned, we also hypothesized that risk aversion and responsible gambling attitude would serially mediate the associations between the two types of empathy and GD (Hypothesis 9). The conceptual model of partial mediations, which summarized all the hypothesized paths among the variables, is presented as Fig. 1.

## Methods

### Participants and procedures

The current study adopted convenience sampling to recruit Chinese lottery gamblers in mainland China (i.e., Chongqing, Leshan, Enshi, Suzhou, and Wenzhou) from November 2021 to February 2022. Potential participants, at public places near lottery sales shops in these five cities, were approached and informed of the purposes of this study and their rights to refuse or withdraw from the study at any time without any negative consequences, by trained research assistants. Then, potential eligible participants (i.e., aged 18 years and above and having bought lottery tickets in the past year) completed an anonymous paper-version questionnaire on site after providing their informed consent to participate in this survey. A small monetary incentive (about 1.5 USD on average) was provided to participants when they completed and returned the questionnaire. Ethical approval of conducting this study was granted by the department of psychology of the affiliated university of the authors (reference number: DPSY2021-21). In total, 714 questionnaires were returned and 580 of them (*M*_*age*_ = 34.07, *SD* = 13.36; 50.4% female) were considered as valid and included for formal data analysis in this study. For these participants excluded in the present study, some of them (*n* = 74) did not meet inclusion criteria (i.e., age ≥ 18 and past-year lottery gamblers), while others (*n* = 60) answered two attention test questions (i.e., “Please circle ‘2’ for this attention test.” and “Please circle ‘5’ for this attention test.”) incorrectly.

### Data collection

The 9-item DSM-5 diagnostic criteria for GD [1], which has been used in Chinese gamblers [42, 50, 51], was used in this study to measure respondents’ susceptibility to GD. Participants indicated whether they had experienced any symptoms of GD (e.g., preoccupation with gambling) during the past year with a dichotomous response (i.e., 0 = *no* and 1 = *yes*). A higher total score indicated a higher susceptibility to GD. In addition, the cutoff score (i.e., ≥ 4), which was consistent with previous studies among Chinese people [51–53], was adopted to estimate the percentage of GD among past-year lottery gamblers. The reliability (i.e., *KR-20*) of this scale was 0.75 in current study.

The 22-item Chinese version [54] of the Interpersonal Reactivity Index [55] was used to assess the two independent types of empathy (i.e., cognitive empathy and emotional empathy). Consistent with previous studies [56, 57], cognitive empathy was measured by the mean score of 11 items, which included the perspective taking subscale (5 items; e.g., “I sometimes try to understand my friends better by imagining how things look from their perspective”) and fantasy subscale (6 items; e.g., “When I am reading an interesting story or novel, I imagine how I would feel if the events in the story were happening to me”), while another 11 items, including 6-item emotional contagion subscale (e.g., “I often have tender, concerned feelings for people less fortunate than me”) and 5-item personal distress subscale (e.g., “When I see someone who badly needs help in an emergency, I go to pieces”), were used to assess emotional empathy. Participants rated all items on a 5-point scale, from 1 = *not appropriate* to 5 = *very appropriate*. A higher mean score indicated a higher level of the corresponding type of empathy. Cognitive empathy and emotional empathy, had McDonald’s ω of 0.76 and 0.67, respectively, in this study.

The 6-item General Risk Aversion Scale [31] was used to measure individuals’ risk aversion, which is a negative general attitude arising from potential risk. Participants rated each item (e.g., “I prefer situations that have foreseeable outcomes”) from 1 = *strongly disagree* to 7 = *strongly agree*. A higher mean score indicated a higher level of risk aversion. Internal consistency was 0.77, measured by McDonald’s ω, in this study.

The 7-item belief subscale of the Chinese version [42] of the Positive Play Scale [41] was used to assess individual’ attitudes toward responsible and controlled gambling. Participants answered all items (e.g., “Gambling is not a good way to make money”) on a 5-point Likert scale (from 1 = *strongly disagree* to 5 = *strongly agree*). The higher mean score represented a higher level of responsible gambling attitude. The McDonald’s ω of this scale was 0.77 in current study.

The participants were asked to report their sex (0 = *male*, 1 = *female*) and age (years). They were also asked about the frequency of their past-year lottery gambling (0 = *never*, 1 = *rarely,* 2 = *sometimes,* 3 = *often,* 4 = *always*), and the participants without past-year lottery gambling experiences (i.e., choosing 0 = *never*) were excluded from the present study.

### Data analysis

Descriptive and correlation analyses were conducted in SPSS 26.0 [58]. Then, the multiple mediation model was tested in the Lavaan package of R, with the full information maximum likelihood estimation method with robust standard errors, which deals with missing and nonnormal data [59, 60]. According to Kline’s recommendation [61], the comparative fit index (CFI; ≥ 0.90), root mean square error of approximation (RMSEA; ≤ 0.08), and standardized root mean square residual (SRMR; ≤ 0.08) were used to evaluate the goodness of fit of our hypothesized multiple mediation model. If the proposed path model did not fit our data well, the alternative model would be tested. Akaike’s information criteria (AIC) and Bayesian information criteria (BIC) would be used to compare the conceptual model and the alternative model, with the superior one bearing smaller values [61]. For mediation testing, the indirect effects were estimated with a 95% confidential interval based on the bias-corrected percentile method with 5,000 bootstrap samples. Statistical significance was accepted at* p* < 0.05 in all analyses.

## Results

### Descriptive and correlation analyses

Statistical descriptions of all participants’ characteristics are shown in Table 1, and the percentage of GD among past-year lottery gamblers was 9.1%. In addition, the correlation coefficients of the psychological and demographic variables of this study are presented in Table 2. Hypothesis 1 was partially supported because GD showed a significant and negative correlation with cognitive empathy (*r* = –0.10, *p* < 0.05) but not emotional empathy (*r* = –0.05, *p* = 0.27). In addition, both cognitive empathy and emotional empathy were positively correlated with risk aversion (*r* = 0.32 and 0.44 respectively, *p* < 0.001), supporting Hypothesis 2. However, no significant correlation was found between risk aversion and GD (*r* = 0.08, *p* = 0.07). Thus, Hypothesis 3 was not supported. Responsible gambling attitude showed a significant and negative correlation with GD (*r* = –0.36, *p* < 0.001) and significant and positive correlations with the two types of empathy (*r* = 0.27 to 0.34, *p* < 0.001), supporting both Hypothesis 5 and Hypothesis 6, respectively. Responsible gambling attitude was also positively associated with risk aversion (*r* = 0.21, *p* < 0.001), supporting Hypothesis 8.

**Table 1:** Means, standard deviations, and frequencies for study variables

Variables	Range/Modalities	n (%)	*M* ± *SD*
Age	18–91		34.07 ± 13.36
Gender	Female (1)	292 (50.4%)	
	Male (0)	287 (49.6%)	
Frequency of lottery gambling during past year	Rarely (1)	293 (49.5%)	
	Sometimes (2)	213 (36.7%)	
	Often (3)	64 (11.0%)	
	Always (4)	10 (1.7%)	
Gambling disorder	Yes (1)	53 (9.1%)	
	No (0)	527 (90.9%)	
Gambling disorder (total score)	0–9		1.01 ± 1.61
Cognitive empathy (mean score)	1.18–4.91		3.38 ± 0.53
Emotional empathy (mean score)	1.91–4.91		3.29 ± 0.46
Risk aversion (mean score)	1–7		4.38 ± 0.90
Responsible gambling attitude (mean score)	2–5		3.93 ± 0.65

**Table 2:** Correlation matrix

	1	2	3	4	5	6	7
1. Cognitive empathy	1						
2. Emotional empathy	0.55^***^	1					
3. Risk aversion	0.32^***^	0.44^***^	1				
4. Responsible gambling attitude	0.34^***^	0.27^***^	0.21^***^	1			
5. Gambling disorder	–0.10^*^	–0.05	0.08	–0.36^***^	1		
6. Sex^a^	0.21^***^	0.37^***^	0.06	0.20^***^	–0.12^**^	1	
7. Age	–0.24^***^	–0.18^***^	0.01	–0.25^***^	0.15^**^	–0.34^***^	1

^*^p < 0*.*05, ^**^ p < 0.01, ^***^ p < 0.001

^a^Binomial variable: 0 = *male*, 1 = *female*

### Path analysis and mediation analysis

**Fig. 2:**
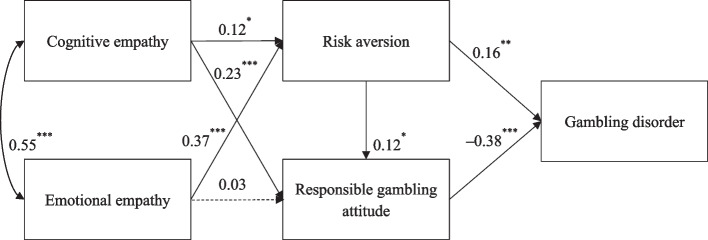
The final path model with standardized estimates. Note: ^*^p < 0*.*05, ^**^ p < 0.01, ^***^ p < 0.001. This model has controlled for sex and age with their significantly correlated variables

The conceptual model (Fig. 1) was tested with path analysis while controlling for the effects of sex and age on the correlated variables based on the bivariate correlation analysis. Its goodness of fit was unsatisfactory, χ^2^ (2) = 14.38, CFI = 0.96, RMSEA = 0.10, 90% CI [0.06, 0.16], SRMR = 0.03. Therefore, an alternative model, which was a full mediation model after removing the two nonsignificant direct paths form cognitive empathy, as well as emotional empathy to GD, was also tested. The resultant path model showed a good fit with the current data, χ^2^ (4) = 13.25, CFI = 0.97, RMSEA = 0.07, 90% CI [0.03, 0.11], SRMR = 0.03. Also, AIC and BIC values of the full mediation model (AIC = 11276.41; BIC = 11411.67) were smaller than those of the conceptual model (AIC = 11280.17; BIC = 11424.14). In this full mediation model (Fig. 2), the standardized coefficients of all hypothesized paths were significant (*p* < 0.05), with the exception of the direct path from emotional empathy to responsible gambling attitude (*β* = 0.03, *p* = 0.62).

The results of mediation analysis using bootstrapping approach are displayed in Table 3. The total indirect effect of cognitive empathy on GD was –0.07 (95% CI [–0.12, –0.03]), whilst the total indirect effect of emotional empath on GD was not significant (*β* = 0.03, 95%CI [–0.02, 0.08]). In addition, the indirect effects of both cognitive empathy and emotional empathy on GD via risk aversion were significant (*β* = 0.02, 95%CI [0.004, 0.04] and *β* = 0.06, 95%CI [0.02, 0.10], respectively), supporting Hypothesis 4. Hypothesis 7 was partially supported because of the significant indirect effect from cognitive empathy to GD via responsible gambling attitude (*β* = –0.09, 95%CI [–0.13, –0.05]); however, the indirect effect from emotional empathy to GD via responsible gambling attitude was nonsignificant (*β* = –0.01, 95%CI [–0.05, 0.03]). Last but not least, risk aversion and responsible gambling attitude serially mediated the effects of two types of empathy on GD (*β* = –0.01, 95%CI [–0.01, –0.001] and *β* = –0.02, 95%CI [–0.03, –0.004], respectively). Thus, Hypothesis 9 was also supported.

**Table 3:** Testing the pathways of the multiple mediation model

Path	*β*	95%CI (lower, upper)	Statistical significance
Total indirect effect of cognitive empathy on gambling disorder	–0.07	(–0.12, –0.03)	Significant
Total indirect effect of emotional empathy on gambling disorder	0.03	(–0.02, 0.08)	Nonsignificant
Indirect effect from cognitive empathy to gambling disorder via risk aversion	0.02	(0.004, 0.04)	Significant
Indirect effect from emotional empathy to gambling disorder via risk aversion	0.06	(0.02, 0.10)	Significant
Indirect effect from cognitive empathy to gambling disorder via responsible gambling attitude	–0.09	(–0.13, –0.05)	Significant
Indirect effect from emotional empathy to gambling disorder via responsible gambling attitude	–0.01	(–0.05, 0.03)	Nonsignificant
Indirect effect from cognitive empathy to gambling disorder via risk aversion and responsible gambling attitude	–0.01	(–0.01, –0.001)	Significant
Indirect effect from emotional empathy to gambling disorder via risk aversion and responsible gambling attitude	–0.02	(–0.03, –0.004)	Significant

## Discussion

This study explored the empathy-GD association based on social cognitive theory [5]. Our findings provided empirical support for applying social cognitive theory to understanding how empathy is associated with GD via attitudinal mediator. To be specific, our data provided preliminary evidence for individuals’ attitudes toward both general risk (i.e., risk aversion) and responsible gambling mediating the negative correlation between empathy GD.

This study also revealed the relatively different relationships between the two types of empathy and GD. Regarding cognitive empathy, both the bivariate and multivariate analyses supported its significant, negative association with GD, despite the small effect size. Consistent with the previous findings of the deficits in awareness of one’s thoughts and feelings in gamblers with GD [62], our results suggests that cognitive empathy, including the awareness of others’ thoughts and feelings, may be a psychological construct in explaining individual differences in GD. Our findings were also in keeping with previous studies highlighting the importance of its role in mental disorders [63, 64]. Regarding emotional empathy, a nonsignificant association (i.e., bivariate correlation, direct effect, and total indirect effect) was found with GD. Our findings provide preliminary support for the potential diverse functions and roles of these two types of empathy, which should be examined as distinct constructs, instead of a unidimensional construct, in future studies of mental disorders, including GD. However, one should also note that the effect size of the association between cognitive empathy and GD link was statistically significant but small, which may restrict the practical implication of such finding. Although cognitive empathy could be nurtured and fostered via biography and role playing [65, 66], the cost-effectiveness of empathy-related programs for GD prevention must be further evaluated in future studies.

This study further contributed to the literature because it was the first to test and show the positive associations between the two types of empathy and risk aversion. These correlations can plausibly be attributed to the attention bias to negative cues among individuals with high levels of empathy [32, 33, 67, 68], as such bias predisposes them to observe and learn others’ negative attitudes towards an event (e.g., risky situations in our case). However, to our surprise, risk aversion had a nonsignificant bivariate correlation with GD. In contrast, the path from risk aversion to GD in our final multiple mediation model was significant and positive, resulting in positive indirect paths from the two types of empathy to GD via risk aversion. The risk-enhancing role of risk aversion to GD in the present study might be attributed to the suppression effect [69], by which the presence of responsible gambling attitude, which was significantly correlated with risk aversion, increased the explanatory power of risk aversion on GD. Future studies are warranted in order to clarify whether and how risk aversion is associated with GD and whether such a suppressor effect exists consistently across ethnics, ages, and socioeconomic groups.

In this study, responsible gambling attitude, which were also positively correlated with both the two types of empathy and risk aversion, was identified as the most salient and proximal factor of GD in the path model. In particular, it significantly mediated the effect of cognitive, rather than emotional empathy, on GD. These findings may be gleaned from the nature of these two empathic components: emotional empathy is an ability to feel others’ emotional states, while cognitive empathy refers to the ability to take others’ perspectives and understand their intentions and attitudes [6]. Although they are expected to work together in facilitating social interaction [11, 12], our current findings suggest that gamblers with higher levels of cognitive empathy are probably more likely to understand and develop responsible gambling attitude. Responsible gambling attitude stresses controlled gambling for entertainment purposes only and is commonly shared by most people (including gamblers’ family and friends) and the government [70, 71], while such attitude may in turn protect them from developing GD [41, 42]. One should also note that, the results of this study also found responsible gambling attitude mediating the association between risk aversion and GD. Such results are in line with the positive correlation between risk propensity and irrational gambling cognition reported in previous research [49]. Moreover, the negative association between cognitive empathy and GD was fully mediated by risk aversion and responsible gambling attitude. These results suggested the potential protective role of attitudinal factors, especially responsible gambling attitude, in lowering one’s risk of GD. In addition to other common approaches (e.g., highlighting the negative consequences of disordered gambling) in public psycho-educational programs to prevent GD, promoting responsible gambling attitude might be considered as a potentially cost-effective alternative in those programs.

Based on social cognitive theory, we proposed and tested a conceptual model with directional paths among the social cognitive variables (i.e., empathy and attitudes) with GD as the outcome variable. However, there were potential alternative models for the correlations found among these variables. For example, individuals with GD tend to show neurological abnormalities in brain regions, including some related to social function [72], and such abnormalities may be the cause of various deficits in social abilities, including the lower levels of both emotional empathy and cognitive empathy. Furthermore, it is a logical speculation that disordered gamblers, who often experience low cognitive and behavioral control over gambling [1, 73], may also report low levels of risk aversion and responsible gambling attitude. However, to the best of our knowledge, there is no theoretical nor empirical support for attitudinal factors (e.g., risk aversion in this study) being the antecedents of empathic abilities. Given we taking the social cognitive perspective to understand GD, we only tested only the proposed conceptual path model, but the cross-sectional design of the present study in fact did not allow for the empirical testing of the causality among the variables. Therefore, the study is warranted to be replicated with longitudinal design to provide data for testing temporal relationships among these variables.

In addition to the cross-sectional design, another limitation of this study warrants caution when interpreting its findings. The generalizability of our findings may be limited because participants in our study were past-year lottery gamblers recruited through convenience sampling near lottery sales shops. One should note that lottery buying imposes less risk of GD when comparing with other types of gambling (e.g., casino and sport gambling) [74]. Despite the percentage of GD gamblers was 9.1% in this study, the prevalence of GD in Chinese gamblers can be estimated only with a more representative Chinese sample recruited by better probability sampling method. Indeed, further studies are warranted to test the replicability of our results, especially regarding the role of responsible gambling attitude in GD, with a more representative sample of gamblers across gambling types/platforms (including online and offline gamblers) as well as GD severity level (e.g., clinical samples).

## Conclusion

The present study is the first to investigate whether two types of empathy are associated with GD via attitudes toward general risk and the specific type of gambling. Under the framework of social cognitive theory [5], its findings advance our understanding of the attitudinal factors linking empathy to behavioral addictions. In line with the theory, our findings provided evidence that attitudinal factors (i.e., risk aversion and responsible gambling attitude) played potential mediating roles in the associations between social abilities (i.e., cognitive empathy and emotional empathy) and addictive behaviors (i.e., GD).

Compared with emotional empathy, cognitive empathy seems to have a mild but statistically significant correlation with fewer GD symptoms, in which risk aversion and responsible gambling attitude fully mediation the correlation. In addition, responsible gambling attitude was not only the most proximal protective factor against GD but also a mediator in the association between risk aversion and GD. Our resultant path model suggests that the cognitive components of social abilities, leading to responsible gambling attitude, may be incorporated into future GD prevention programs.

## Data Availability

The dataset generated and/or analyzed in the current study is not publicly available, but is available from the corresponding author on reasonable request.
